# A meta‐ethnography of the facilitators and barriers to successful implementation of patient complaints processes in health‐care settings

**DOI:** 10.1111/hex.12645

**Published:** 2017-11-07

**Authors:** David A. H. Scott, Suzanne M. Grant

**Affiliations:** ^1^ Bristol Dental School University of Bristol Bristol UK; ^2^ Population Health Sciences University of Dundee Dundee UK

**Keywords:** meta‐ethnography, patient complaints, patient dissatisfaction, patient perspectives, professional perspectives, qualitative research synthesis

## Abstract

**Objective:**

To synthesize experiences of the patient complaints process for patients and health‐care professionals to identify facilitators and barriers in the successful implementation of patient complaints processes. This will assist the development of cultural change programmes, enabling complaints managers to incorporate stakeholder perspectives into future care.

**Design:**

Systematic literature search and meta‐ethnography, comprising reciprocal syntheses of “patient” and “professional” qualitative studies, combined to form a “line‐of‐argument” embodying both perspectives.

**Data sources:**

MEDLINE, CINAHL and PsycINFO (database inception to April 2015) were searched to identify international literature in primary and secondary health‐care settings, involving qualitative data collection and analysis. Further studies were identified from hand‐searching relevant journals, contacting authors, article reference lists and Google Scholar.

**Results:**

A total of 13 papers, reporting 9 studies from the United Kingdom, Sweden, Australia and New Zealand, were included in the synthesis. Facilitators and barriers to the successful implementation of patient complaints processes were identified across the perspectives of both patients and health‐care professionals. Patients sought to individualize the complaints process by targeting specific professionals who engaged in practices that undermined the identity of patients. In contrast, professionals obscured their own individualism through maintaining a collective identity and withholding personal judgement in relation to patient complaints.

**Conclusions:**

Complainants recognized health‐care professionals as bearers of individual accountability for unsatisfactory care, in opposition to the stance of collective responsibility endorsed by professionals. Implementation of patient complaints processes must reconcile the need for individualized resolution, whilst striving to improve the future provision of health care through a collaborative approach between patients and professionals.

## 1 INTRODUCTION

Over the past decade, the volume of complaints made by patients against health‐care professionals in the UK National Health Service (NHS) has risen significantly. For example, the volume of enquiries from members of the public received by the General Medical Council has risen from 3615 in 2007 to 6547 in 2015.^1^, ^2^, ^3^ Similarly for the General Dental Council, the total number of “fitness to practise” complaints rose from 949 in 2007 to 3099 in 2014,^4^, ^5^ with a 31% increase from 2012 to 2013.^6^

The handling of patient complaints by health‐care professionals in the NHS is often presented by managers as an opportunity to improve the quality and safety of future health‐care services.^7^, ^8^ In particular, the “local resolution” of complaints by front‐line clinical staff (eg, general practitioners [GPs], dentists, hospital consultants, nurses) is championed as helping to prevent individual small‐scale issues developing into more serious concerns. This approach has become a fundamental element of the guidance literature for NHS complaints management in the United Kingdom. For instance, the Parliamentary and Health Service Ombudsman (PHSO) has developed a “complaints handling framework” that highlights service improvements as a key outcome of the complaints process.^9^ Yet, while patient complaints are considered an important mechanism through which to better understand and improve patient care, in practice many local complaints are escalated into “fitness to practise” claims against professionals. This has been driven by various social, political and cultural factors, including heightened public awareness of regulatory bodies from press coverage of malpractice claims.^10^

Lloyd‐Bostock and Mulcahy^11^ define the patient complaint as an act by which health‐care professionals are held to account for violating patients’ normative expectations of care. As such, the complaints process can be seen as fundamentally dependent on the underlying social and organizational context. Mulcahy^12^ considers “local resolution” to be a historic remnant of professional self‐regulation and clinical autonomy, responsible for excluding lay and managerial influences from complaints handling. Similarly, Nettleton and Harding^13^ argue that both professional self‐regulation and the new managerialism obstruct complaints processes by reinforcing the control of professionals and managers, respectively.

Research remains limited as to which barriers and facilitators influence the successful implementation of patient complaints processes. Most studies on patient complaints have involved categorizing formal written complaints, rather than investigating the reasons why some informal complaints fail to be resolved when they first arise.^14^ In contrast, qualitative research on the early stages of the complaints process can enlighten our understanding of the informal ways in which patient complaints occur and, in some cases, escalate beyond local resolution. The aim of this meta‐ethnography was to synthesize the views of both patients and health‐care professionals to identify facilitators and barriers in the successful implementation of patient complaints processes.

## 2 METHODS

### 2.1 Eligibility criteria

The systematic search aimed to identify all studies that investigated the experiences of patients and/or health‐care professionals during the patient complaints process, published from database inception to April 2015. Papers were included if they were published in English and involved qualitative data collection and analysis. Studies that primarily focussed on litigation or satisfaction surveys were excluded. No restrictions were placed on publication date or country to provide a synthesis with international relevance, informed by recent political and social changes.

### 2.2 Search strategy

Full searches of the literature were conducted in April 2015 using 3 electronic databases: MEDLINE, CINAHL and PsycINFO. Whereas additional databases were initially considered, the final decision was justified by Toye et al^15^ concluding that 95% of the 60 studies included in their meta‐ethnography were identified from only 3 databases. The searches retrieved articles containing one or more of the following words, drawn from the range of terms used to depict the patient complaints process in published literature: “malpractice,” “complaints,” “grievances,” “negligence” and “dissatisfaction.” Medical Subject Headings (MeSH) and free‐text terms were combined to form a complex search strategy. The grey literature was searched using Google Scholar, utilizing the “cited by” function to identify subsequent studies that had cited those included from the database search. This was followed by hand‐searching relevant journals, a search of the reference lists of papers included from the database search, and additional contact with primary authors of included articles to identify manuscripts in press.

### 2.3 Study selection

**Figure 1:**
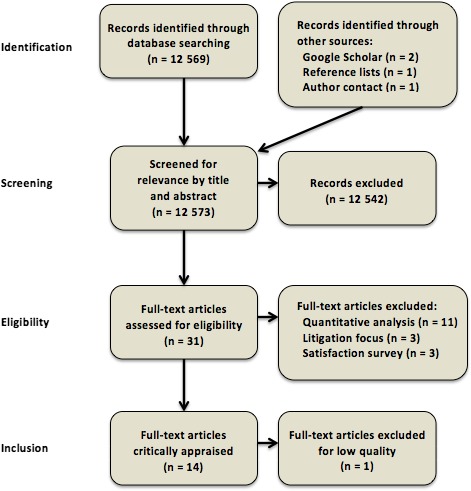
Search process

The literature search followed the Preferred Reporting Items for Systematic reviews and Meta‐Analyses^16^ (PRISMA) format: identification, screening, eligibility and inclusion (Figure 1). The lead author (DS) screened the titles and abstracts of articles retrieved by database searching and other sources for relevance. Of those articles judged potentially relevant, full‐text copies were located and assessed for inclusion in discussion with the second author (SG). This process aimed to comprehensively identify all published studies that met the inclusion criteria, using a systematic and replicable procedure.

### 2.4 Critical appraisal

A critical appraisal of the included articles was conducted to ensure that the findings were based on substantial empirical evidence and had been verified by a robust analysis. The process was based upon an eight‐question version of the Critical Appraisal Skills Programme (CASP) qualitative research checklist,^17^ modified by the specifically meta‐ethnographic criteria of conceptual clarity and interpretive rigour proposed by Toye et al^18^ (Table 1). DS critically appraised all studies and assigned each study a numerical score out of 8, after which SG independently appraised a cross‐section of studies rated as “low” (0‐2), “moderate” (3‐5) and “high” (6‐8) quality to check for consistency. Disagreement initially centred on the clarity of the research aims and concepts stated by 2 studies both judged to be of “low” to “moderate” quality; following further discussion, however, consensus was reached by only excluding the lowest‐quality study. The 2 studies ranked with the highest scores, 1 “patient” study and 1 “professional” study, were used as “index” studies and acted as the first studies from which concepts were translated into other studies, thereby shaping the analysis.^19^

**Table 1:** Critical appraisal questions based on a modified version of Critical Appraisal Skills Programme checklist

Question
Was there a clear statement of the aims of the research?
Has the sample population been defined?
Has the relationship between researcher and participants been adequately considered?
Have ethical issues been taken into consideration?
Has the interpretation been challenged?
Have contradictory data been taken into account?
Are the concept‐indicator links clear?
Are the concepts clear and readily translatable?

### 2.5 Synthesis of findings

The technique of meta‐ethnography was selected for synthesizing the findings of the included studies. This method of synthesis was chosen over alternative approaches as it has been found to be more suitable for the development of analytical, rather than descriptive, findings (cf. thematic synthesis).^20^ Meta‐ethnography relies on a process of “translation,” whereby concepts from one study are introduced into another and assessed for the extent to which they can account for a perceived phenomenon within a different context.^21^ Three outcomes of translation are possible: (i) “reciprocal” translations of accounts that are analogous, (ii) “refutational” translations of accounts that are contradictory, and (iii) “line‐of‐argument” translations of accounts that interpret different aspects of the same phenomenon, ultimately producing a whole that is greater than the sum of its individual parts.^22^

For each of the included articles, data on the design, analysis and key concepts were extracted and recorded by DS. The synthesis process comprised 3 stages: (i) a reciprocal translation of the “patient” studies to understand service users’ responses to experiencing dissatisfying health‐care encounters and their perceptions of making complaints; (ii) a reciprocal translation of the “professional” studies to comprehend health‐care professionals’ views on receiving, and their perceptions of handling, patient complaints; and (iii) a line‐of‐argument translation of all studies to outline how complainants’ and professionals’ differing perspectives of health‐care disputes influence the local resolution of complaints and future provision of services. A refutational translation was initially considered instead of a line‐of‐argument translation, but it became apparent during synthesis that concepts from “patient” and “professional” studies were not strictly contradictory in nature and more accurately described alternate perspectives of the same phenomenon.

The key concepts, or “second‐order constructs” (ie, interpretations made by the authors of the included studies), were extracted by DS and recorded in a Microsoft Excel spread‐sheet, alongside illustrative quotations from study participants (“first‐order constructs”).^23^ The context and meaning of second‐order constructs were preserved by maintaining the authors’ own terminology and definitions. Using an approach that resembles grounded theory's constant comparative method, different concepts were compared for similarities and contradictions, leading to the overriding adoption of existing concepts or the generation of new concepts that provided a fuller account of a given phenomenon and resolved any contradictions.^24^ This was performed by systematically and sequentially comparing concepts using recorded study characteristics (ie, publication date, country, health‐care setting, sample size, recruitment method, age range and gender ratio of participants, and method of data analysis) as context for the comparisons. Juxtaposition of both the first‐ and second‐order constructs led to the development of original “third‐order constructs” by the authors (DS and SG), comprising a new understanding of the phenomena under study. For example, the second‐order construct “dehumanization” more fully explained specific instances of objectifying behaviour than being “treated with disrespect,” and was therefore adopted as the third‐order construct “objectification.” Whereas “changing clinical practice,” such as the provision of a more limited service, initially appeared incompatible with “overinvestigating”—a contradiction that could be explained by the new third‐order construct “withholding personal judgement.”

## 3 RESULTS

### 3.1 Study characteristics

Nine studies were identified that met the inclusion criteria and passed the critical appraisal process (Table 2). These were reported within 13 articles published between 1998 and 2015 and involved 195 participants (99 patients and 96 professionals). Studies were included from a relatively diverse range of high‐income countries: 2 in Sweden,^28^, ^31^, ^32^ 2 in Australia,^29^, ^30^, ^33^ 2 in New Zealand^36^, ^37^ and 3 in the United Kingdom.^25^, ^26^, ^27^, ^34^, ^35^ Each of the studies focussed on an individual country, and no study has yet examined the patient complaints process from an international perspective. The following sections comprise reciprocal translations of the “patient” and “professional” studies, prior to a line‐of‐argument translation of all studies.

**Table 2:** Study characteristics

Author(s)	Year	Country	Health‐care setting	Sample size	Participants	Age range	M:F ratio	Method of data analysis
Coyle^25^ (index study)	1997	UK	Mixed	41	Theoretical sample of health service users selected through a household survey	18‐79 y	20:21	Grounded theory
Coyle^26^	1999							
Coyle^27^	1999							
Eriksson and Svedlund^28^	2007	Sweden	Secondary	6	Convenience sample of hospital patients identified through a patients’ advice group	29‐59 y	2:4	Latent content analysis
Howard^29^	2011	Australia	Secondary	16	Convenience sample of hospital patients invited through media advertisements	18‐79 y	5:11	Phenomenology
Howard, Fleming and Parker^30^	2013							
Skär and Söderberg^31^	2012	Sweden	Mixed	23	Purposive sample of patients identified through a patients’ advice group	18‐76 y	9:14	Qualitative content analysis
Söderberg, Olsson and Skär^32^	2012							
Finney Lamb et al^33^	2008	Australia	Secondary	23	Purposive sample of opioid‐dependent women and staff at an opioid treatment service	Not specified	Not specified	Thematic analysis
Allsop and Mulcahy^34^	1998	UK	Secondary	35	Representative sample of hospital consultants selected through a postal survey	Not specified	Not specified	Grounded analysis
Jain and Ogden^35^ (index study)	1999	UK	Primary	30	Representative sample of general practitioners selected through a postal survey	Not specified	16:14	Frame analysis
Cunningham and Dovey^36^	2006	New Zealand	Secondary	12	Convenience sample of hospital‐based specialist doctors identified through a medico‐legal society	Not specified	Not specified	Inductive analysis
Stuart and Cunningham^37^	2015	New Zealand	Primary	9	Convenience sample of dentists invited through professional networks	Not specified	5:4	Phenomenology

### 3.2 Reciprocal translation of “patient” studies

Reciprocal translation of the key concepts extracted from the 5 “patient” studies synthesized three third‐order constructs: “objectification,” “negative stereotyping” and “abnegating responsibility” (Table 3).

**Table 3:** Reciprocal translations of “patient” and “professional” studies

Third‐order construct	Second‐order construct	Original description
“Patient” studies		
Objectification	Dehumanization^26^	“People who were unhappy with their care felt they had been treated as ‘non persons’ and that little recognition was given to them as human beings.” (p. 107)
	Treated with disrespect^29^	“A sense that the participants were made to feel insignificant and, on many occasions, felt that they were being treated with disrespect.” (p. 146)
Negative stereotyping	Stereotyping^26^	“Practitioners routinely categorise patients according to their subjective judgements about patients’ characteristics and behaviour.” (p. 110)
	Treated with disrespect^29^	“A sense that the participants were made to feel insignificant and, on many occasions, felt that they were being treated with disrespect.” (p. 146)
	Not being respected as a person^31^	“The body language and facial expressions of the professionals showed that they did not respect them as individuals.” (p. 282)
	Feelings of being troublesome^28^	“Participants feel that they are troublesome and have become the type of patient they do not want to be.” (p. 442)
	Anticipation of not being believed^33^	“Women reported that they believed that health staff would not take them seriously or believe them if they made a complaint about health care because they used drugs.” (p. 69)
Abnegating responsibility	No one takes responsibility^28^	“Caregivers refuse to talk to participants, something they believe is because of the fact that those involved do not want to take the consequences for their decisions.” (p. 441)
	Inconsistent care^29^	“Each participant made reference to the standards of care not being appropriate, consistent, or adequately meeting their needs in some respect.” (p. 150)
	Left without a personal excuse^32^	“It would have been easier for them to proceed if they had instead received a personal excuse from the healthcare personnel who had treated them badly, rather than a letter from the head of the clinic.” (p. 147)
“Professional” studies		
Purposive categorization	Typifications^34^	“[A] major way in which doctors accounted for complaints was to attribute them to the character of the complainant or lay person.” (p. 814)
	Volatile clients^33^	“Staff reported that they used their knowledge of different clients to decide what information to ignore and what information to respond to.” (p. 70)
	Problem patients^36^	“Respondents indicated actively attempting to identify likely complainants, based on their sense (and that of their staff) of the quality of the doctor‐patient relationship.” (p. 5)
Withholding personal judgement	Changes in practice^37^	“[Dentists] report being more aware of record‐keeping and of informing patients about what they were doing, particularly in ‘wait and watch’ situations.” (p. 29)
	Changing clinical practice^35^	“Some [GPs] reported having changed their clinical practice as a result of the complaint such as offering a more limited service.” (p. 1598)
	Overinvestigating^36^	“Doctors interpreted this form of positive defensive practice as disadvantageous to patients and the health system generally, but were aware of the utility of over‐investigating as a response to societal pressure for certainty, and as a defence mechanism, should a complaint occur.” (p. 5)
Maintaining professional identity	Professional networks^34^	“Help seeking was a form of protection, as the individual could talk to others who shared the same framework of meaning and knowledge base.” (p. 817)
	Relationships^35^	“Participants also described the effect on the practice where they worked… Some participants described how their relationships had improved because of the complaint.” (p. 1598)

#### 3.2.1 Objectification

Patients described situations during the course of their treatment in which they felt that they were treated by professionals as inanimate objects, rather than human beings worthy of dignity and respect. The standard of care was considered by patients to be unacceptable on this basis, prompting them to complain. Coyle^26^ defined the concept of “dehumanization” as “a sense of being treated as an object on a highly mechanised and routinized medical production line” (p. 107). It is notable that 5 of the 21 women interviewed by Coyle used this metaphor of a “production line” in their own experiences of childbirth. Such a viewpoint was corroborated by Howard^29^ who demonstrated clinical scenarios in which participants depicted their treatment by health professionals as if they were “a lab animal,” “a guinea pig” or “a toy” for testing out the hospital's new equipment. As a 55‐year‐old male patient related: “I just wanted them to acknowledge that people are people; people in their care are actual real human bodies and not just pieces of meat that you can shove around to your heart's content.”^29^

#### 3.2.2 Negative stereotyping

Patients, particularly women, those from ethnic minorities and working‐class men, frequently reported that health professionals categorized them as a certain type of patient and managed their medical care on the basis of superficial judgements. These assumptions were unanimously negative and were often related to perceptions of low intelligence, childishness, dishonesty, idleness or psychological maladjustment.^26^ Such stereotyping was uniformly present across professions (eg, doctors and nurses) and both primary and secondary care services (eg, general medical practices and opioid treatment services). Participants variously reported their experiences of being negatively “labelled,”^26^ as having “got a name,”^32^ or simply being thought of as “one of those patients”^28^ and that their health‐care concerns were disregarded by professionals as a consequence. In the process of complaining about the standard of care they had received, patients recounted a fear of becoming further stereotyped as a “troublesome patient,” acting as an additional disincentive to following through the complaint.^28^

#### 3.2.3 Abnegating responsibility

Patients expected individual health‐care professionals to maintain a sense of personal duty, ensuring that the expected standards of a patient's care were met. However, as Eriksson and Svedlund^28^ recognized, from the standpoint of many patients, professionals “[did] not want to take the consequences for their decisions” (p. 441). This attribute was found across professional groups and organizational settings. For example, a patient was repeatedly referred between a GP and a social worker, neither of whom was willing to take responsibility for completing the required occupational health assessment. Similarly, in the study by Howard,^29^ another patient expressed concern over the lack of continuity in his care, in that he “didn't see the same nurse twice” and felt it to be “a case of the blind leading the blind.” Söderberg et al^32^ found that patients were particularly critical of the perceived managerial view of health care as “a closed system where no one does anything wrong when routines are followed” (p. 147). In marked contrast, patients expected health‐care professionals to be individually accountable for their actions, rather than blaming “the system.”

### 3.3 Reciprocal translation of “professional” studies

Reciprocal translation of the key concepts extracted from the 5 “professional” studies synthesized three third‐order constructs: “purposive categorization,” “withholding personal judgement” and “maintaining professional identity” (Table 3).

#### 3.3.1 Purposive categorization

The categorization of patients as a means to inform the prescription of clinical care has been co‐opted within the non‐clinical territory of handling patient complaints. Allsop and Mulcahy^34^ outline the attribution of complaints by hospital consultants to the character of the complainant in what they refer to as “typifications,” consequently labelling complainants as “moaners,” “abusers” and “malcontents.” Cunningham and Dovey^36^ extend this process of categorization still further with hospital doctors’ attempts to pre‐empt future complaints through the identification of “problem patients.” Such a response involved a collaborative interprofessional approach in “actively attempting to identify likely complainants, based on their sense (and that of their staff) of the quality of the doctor‐patient relationship.” This purposeful instance of categorization allowed professionals to act in such a way that they could successfully counter any allegations of negligence. Likewise, Finney Lamb et al^33^ reported that nurses categorized some complainants as “volatile,” in that they were more likely to make complaints due to their overemotional characters, thereby informing appropriate consideration or dismissal of the complaint.

#### 3.3.2 Withholding personal judgement

One of the consequences of receiving a complaint was that health‐care professionals felt their claim to the patient's trust through respect for expert judgement had been undermined. This resulted in a withholding of personal judgement in the provision of patient care, which was evidenced through a number of defensive strategies utilized by hospital consultants, GPs and dentists to protect themselves from future complaints. These changes included a self‐defensive repetition of standardized warnings,^37^ the decision not to persuade patients against desired but unnecessary treatments^35^ and the provision of superfluous investigations.^36^ In some situations, this overcompensatory behaviour was judged by professionals to be inimical, rather than beneficial, to a patient's well‐being. For example, a paediatrician summarized how the indiscriminate application of medical investigations may adversely affect patients’ care: “I think I actually expose kids to risk more… not only will I spend money, health dollars, on testing, but I will also put kids through painful and potentially risky procedures to satisfy parental concern.”^36^ In contrast, professional overcompensation was conspicuously absent from the accounts of nurses; an omission that might have been due to a lack of clinical autonomy in treatment decisions or a perceived lesser risk of patient complaints.

#### 3.3.3 Maintaining professional identity

Health‐care professionals frequently relied upon professional networks for moral support in the event of a patient complaint. This reaction could be interpreted as an attempt to collectivise responsibility and minimize individual accountability. Allsop and Mulcahy^34^ inferred that “complaints provide an opportunity for group interaction and the demonstration of solidarity as well as providing a sense of belonging” (p. 817). The exclusivity of a profession and its sole claim to specialized knowledge provide a barrier to external scrutiny of practice: “You can be light hearted with medical colleagues in a way which wouldn't be understood by outsiders.”^34^ In a similar vein, Jain and Ogden^35^ concluded that having supportive medical colleagues could transform the patient complaints process into a favourable experience that redistributed the weight of personal accountability amongst the practice. These studies demonstrate the beneficial aspects of belonging to an autonomous profession, as is the case for medical practitioners, yet it remains unclear whether similar networks are available for nursing professionals (Table 4).

**Table 4:** Participant quotations from “patient” and “professional” studies

Third‐order construct	Participant quotation
“Patient” studies	
Objectification	“You're just a matter of a number or a bit of file, that's all you are, you're not a certain person. Whereas, once upon a time you'd go to the surgery and as soon as you walked into the doctors you became a human being and he was going to talk to you as one. Now, he's looking at the file all the time, he's not even bothered whether he looks at you…”^25^ (p. 171)
	“…I walked in the door it was almost like I was an experimental object they talked over me, they talked around me the only thing they didn't do was actually talk to me there was no explanation of what I was there for they read my referral and read that I had pain in my shoulder, but there was no interaction with me as a subject…”^29^ (p. 146)
Negative stereotyping	“I felt I was being labelled as being over anxious because I would take him (baby son) there, and say he's been wheezing, or he's been rattling. And they would say something like, they weren't actually listening to what I was saying. I was saying that there is something quite seriously wrong with him, and they weren't paying any attention to me.”^26^ (p. 112)
	“Once you've got a name as being a drug user, it doesn't matter what you say, no one is believing you or listening to you, and I also found the more fuss you make the worse it looks for you. If you start yelling or ranting and raving it's like oh, she's off her face, she's an uncontrollable drug user, we expected this from her.”^33^ (p. 69)
Abnegating responsibility	“…Well I didn't see the same nurse twice so in my opinion no‐one really knew whether I was getting worse or better. I didn't see the same nurse ever, so there was no continuity and I felt that sometimes it was a case of the blind leading the blind…”^29^ (p. 245)
	“It was the wrong person who said I'm sorry… It should have been the person that treated me badly not the person in charge… the excuse should have been more personal.”^32^ (p. 147)
“Professional” studies	
Purposive categorization	“Some of them are very volatile and every day can be a new drama or complaint, and next day it will be fine. Whereas another person it's the exception to get a complaint from them.”^33^ (p. 70)
	“The complaining type… They shake hands with you but they are vicious. Basically, they want you to know they are in charge.”^34^ (p. 815)
Withholding personal judgement	“I would visit at the drop of a hat. I wouldn't try to advise over the phone because I was just too scared of what would ensue if I advised over the phone. If there was a hint that antibiotics were a possibility I'd give them. I wouldn't try and educate the patient out of having their antibiotics.”^35^ (p. 1599)
	“With patients who have ‘watches’ on their teeth, I tell them every single time I see them now. So that they know that I'm keeping an eye on a tooth which may have had a wee R2 lesion on it for 20 years.”^37^ (p. 29)
Maintaining professional identity	“The way the practice handled it, which I think is very good, is that they have a system whereby they believe that if there's a complaint made then it's made against the whole practice.”^35^ (p. 1598)
	“You get support in a semi‐joking way. You can be light hearted with medical colleagues in a way which wouldn't be understood by outsiders. We share the same sense of humour and it may sound sick, but it's a way of managing stress.”^34^ (p. 817)

### 3.4 Line‐of‐argument translation of all studies

**Figure 2:**
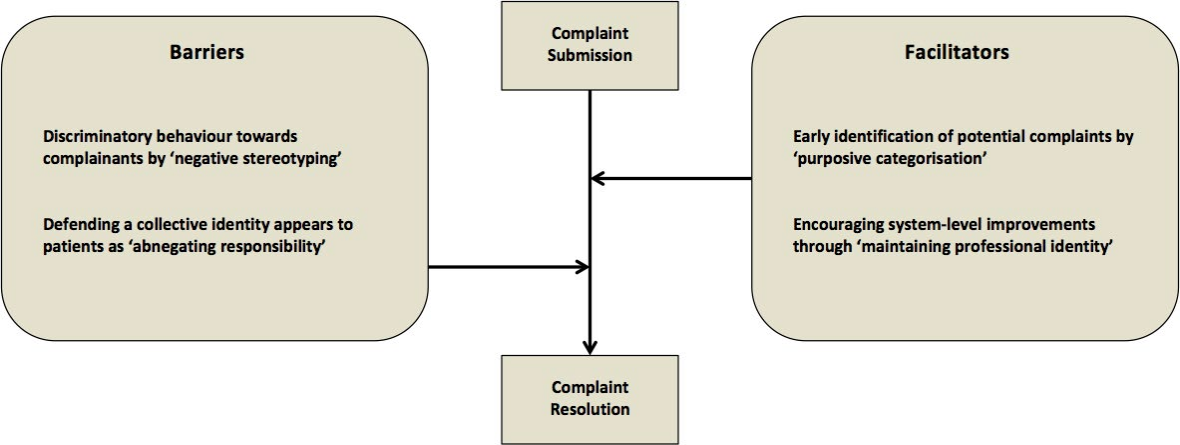
Facilitators and barriers to the successful implementation of patient complaints processes

Line‐of‐argument translation of the six third‐order constructs synthesized from the “patient” and “professional” studies contributed to the identification of facilitators and barriers to the successful implementation of patient complaints processes (Figure 2). The patient complaints process was characterized by a complex route of progression that did not always result in successful resolution. Dissatisfaction with care could be professionally validated^27^, ^28^, ^33^ (eg, through a second expert opinion) or incited^34^, ^35^, ^37^ (eg, through the interference of relatives and friends), leading to a formal complaint. Alternately, the complainant could retract their formal complaint due to disempowerment^26^, ^31^, ^33^ (eg, through the emotional exhaustion of making a complaint), or progress to the successful outcome of resolution^28^, ^33^, ^35^ (eg, through an authentic apology or reparative action).

Throughout the complaints process, the transition from submitting a complaint to achieving successful resolution was mediated by a range of facilitators and barriers. The categorization of specific patients as prone to complaining, while enabling the pre‐emption of future complaints, further replicated the behaviour of negative stereotyping that first led to dissatisfaction, acting as a subsequent barrier to resolution. Similarly, by appealing to a collective professional identity, health‐care professionals facilitated a system‐level approach to improving future services, whereas the same attitude was perceived by patients as obscuring personal responsibility for care.

## 4 DISCUSSION

This study has highlighted the impact of both societal and organizational changes on the relationship between patients and health‐care professionals, particularly with regard to professional autonomy. Key characteristics of professional autonomy include expert knowledge and practice;^38^ self‐evaluation of performance and care;^39^ and control over the nature and volume of medical tasks.^40^ Beardwood et al^41^ claim that weakening of professional autonomy has led to individualization of patient complaints. They cite how representatives of professional nursing bodies in Canada have reacted by concentrating on the provision of legal advice, including the strategic use of apologies in a legal context (ie, nurses are expected to offer their sympathy without incurring personal liability). Concurrently, diminishing professional autonomy has occurred alongside the standardization of clinical care. Armstrong^42^ writes that the emergence of external “decision support” mechanisms (eg, clinical guidelines) has been responsible for re‐focussing professionals’ attention away from individual accountability towards more standardized approaches in the delivery of health‐care services.

As patients are increasingly empowered to critique professional work, health‐care professionals have adopted more defensive strategies to maintain their professional autonomy. Across the varied accounts given by both groups, there was an implicit conflict in the attempts of professionals to depersonalize and standardize complaints resolution, and patients’ perceptions that professionals were attempting to avoid personal blame and recrimination. This raises a significant concern that the current rise in “fitness to practise” claims is to some extent contributing to depersonalization of the complaints process, hindering individualized resolution. While patients were highly attentive to individual professional accountability in personalizing their complaint as much as possible, professionals actively resisted individual blame through the adoption of defensive strategies that drew on the wider socio‐technical system, including the use of professional networks and the repetition of standardized warnings. This juxtaposition of individual vs system‐wide understandings of safety and error is reflected in the work of Reason's “systems approach” to safety, where health‐care organizations are understood as risk‐prone complex systems in which blame cannot be attributed to a single individual.^43^

The validity of a meta‐ethnography's findings is inevitably limited by the breadth and quality of included studies. All 9 studies were reported in high‐income countries, indicating that the findings may not be applicable to low‐ and middle‐income countries. Since completing this meta‐ethnography, qualitative interviews and focus groups in Nepal have identified that barriers to complaints resolution are more often characterized by procedural inadequacies in the complaints system and a heightened power imbalance between service users and providers.^44^ Due to the limited volume and broad diversity of included studies (eg, the Swedish studies were restricted to the patient's perspective; the New Zealand studies only considered professionals’ views; the Australian studies were based entirely in secondary care settings), we were unable to draw substantiated generalizations in the differences between countries or health‐care settings. Lee et al^45^ promote interdisciplinary collaboration as an essential prerequisite for conducting a meta‐ethnography, to ensure the credibility of findings to a wider audience. In this study, the lead researcher was a practising dentist with direct experience of front‐line complaints handling; in turn, the clinical perspective was complemented by methodological and interpretive input from an experienced medical anthropologist. However, presentation of the study's findings to a panel of patient representatives or policy makers may have provided a more robust evaluation of validity.

Current guidance on the handling of patient complaints proposes the need to implement a culture that takes a positive attitude towards complaints, encouraging and welcoming them, while also learning from them to improve the future provision of health‐care services.^8^, ^9^ We present facilitators and barriers to the successful implementation of patient complaints processes that may be used to design new programmes for cultural change. Such a programme should operate on both a person‐ and system‐level: front‐line clinical staff should be encouraged to take accountability for complaints handling, assist potential complainants in determining the form by which they wish their concern to be managed (eg, as feedback or a formal complaint), and participate in a transparent process whereby system‐level strengths and challenges are acknowledged and understood, with context‐specific solutions identified. It would therefore be desirable that future measures of success in complaints handling evaluate both personal resolution and system improvements as final outcomes of the complaints process.

## CONFLICTS OF INTEREST

None declared.
